# Targeting Metabolic and Epigenetic Vulnerabilities in Glioblastoma with SN-38 and Rabusertib Combination Therapy

**DOI:** 10.3390/ijms26020474

**Published:** 2025-01-08

**Authors:** Jennifer Chiou, Valeria Impedovo, Yen Bao Huynh, Ruggiero Gorgoglione, Luiz O. F. Penalva, Alessia Lodi, Andrew J. Brenner, Stefano Tiziani

**Affiliations:** 1 Department of Nutritional Sciences, College of Natural Sciences, The University of Texas at Austin, Austin, TX 78712, USA; jennifer.chiou@austin.utexas.edu (J.C.); valeriaimpedovo@utexas.edu (V.I.); yen.hby@utexas.edu (Y.B.H.); rugorgo@gmail.com (R.G.); alessialodi@utexas.edu (A.L.); 2 Dell Pediatric Research Institute, Dell Medical School, The University of Texas at Austin, Austin, TX 78723, USA; 3 Children’s Cancer Research Institute, UT Health San Antonio, San Antonio, TX 78229, USA; penalva@uthscsa.edu; 4 Department of Cell Systems and Anatomy, UT Health San Antonio, San Antonio, TX 78229, USA; 5 Mays Cancer Center, UT Health San Antonio, 7979 Wurzbach Road, San Antonio, TX 78229, USA; brennera@uthscsa.edu; 6 Department of Oncology, Livestrong Cancer Institutes, Dell Medical School, The University of Texas at Austin, Austin, TX 78723, USA; 7 Department of Pediatrics, Dell Medical School, The University of Texas at Austin, Austin, TX 78723, USA

**Keywords:** glioblastoma, high-throughput drug screening, metabolomics, SN-38, rabusertib, epigenetics, liquid chromatography–high resolution tandem mass spectrometry, subcellular metabolism

## Abstract

Glioblastoma (GBM), the most prevalent primary malignant brain tumor, remains challenging to treat due to extensive inter- and intra-tumor heterogeneity. This variability demands combination treatments to improve therapeutic outcomes. A significant obstacle in treating GBM is the expression of O^6^-methylguanine-DNA methyltransferase, a DNA repair enzyme that reduces the efficacy of the standard alkylating agent, temozolomide, in about 50% of patients. This underscores the need for novel, more targeted therapies. Our study investigates the metabolic–epigenetic impact of combining SN-38, a novel topoisomerase inhibitor inducing DNA double-strand breaks, with rabusertib, a checkpoint kinase 1 inhibitor. We identified this synergistic combination through high-throughput drug screening across a panel of GBM cell lines using a cancer drug library combined with SN-38. A secondary metabolic screening with the PEDS algorithm demonstrated a synergistic modulation of purine, one-carbon, and redox metabolism. Furthermore, the combined treatment led to the significant depletion of epigenetically relevant metabolites such as 5-methyl-cytosine, acetyl-lysine, and trimethyl-lysine. Reduced intermediates of the glutathione cycle indicated increased cellular stress following combinatorial treatment. Overall, the combination of SN-38 and rabusertib synergistically disrupts metabolites associated with epigenetic adaptations, leading to cytotoxicity independent of O^6^-methylguanine-DNA methyltransferase status, thereby underpinning this combination as a promising candidate for combinatorial therapy in GBM.

## 1. Introduction

Glioblastoma (GBM) is the most common and aggressive primary malignant brain tumor, accounting for 49.1% of all central nervous system cancers [1]. Effective treatment of GBM remains challenging, partly due to the blood–brain barrier (BBB), which limits therapeutic infiltration, and partly due to the genetic and epigenetic heterogeneity of GBM cells [2,3,4]. Standard of care therapies for GBM include surgical resection, radiotherapy, and treatment with the alkylating agent temozolomide (TMZ). TMZ, often combined with radiotherapy, induces DNA damage by transferring alkyl groups to nucleotides [5,6]. Despite these therapies, the 5-year relative survival rate for GBM patients in the United States has remained at only 3–6% since 2004, highlighting the limited progress in treatment efficacy since TMZ’s approval for glioblastoma in 2008 [1,5,6,7,8].

TMZ, and earlier agents like nitrosoureas lomustine and carmustine, all initiate DNA damage, ultimately leading to cell death when cells are unable to repair the damage at the G2/M cell-cycle checkpoint [9,10]. The effectiveness of TMZ is modulated by the DNA repair gene O^6^-methylguanine-DNA methyltransferase (MGMT), where the epigenetic silencing of MGMT serves as a favorable prognostic marker, correlating with extended survival and delayed progression in GBM patients [11]. However, only around 50% of the GBM patient population shows silencing of MGMT. For these reasons, methylated MGMT status is now used as a predictor of TMZ treatment sensitivity [12,13,14,15,16]. In addition to MGMT methylation status, post-translational modifications (PTMs) of histones have emerged as crucial prognostic biomarkers. Histone demethylation is implicated in the GBM tumor microenvironment, influencing treatment efficacy and contributing to resistance to TMZ and radiation [17,18]. Therefore, it is not surprising that PTMs play a significant role in GBM patient survival [19].

Altered cellular metabolism is a hallmark of cancer, including GBM, where metabolic reprogramming supports rapid cell growth and survival in nutrient-deprived, hypoxic conditions [20,21,22]. This metabolic flexibility makes glioblastoma particularly resilient and challenging to treat [21]. Metabolomics and metabolic flux analysis have become essential for understanding these shifts, providing insights into the specific pathways GBM cells rely on [23,24]. By measuring metabolite levels and nutrient flux, these tools reveal metabolic dependencies that can be targeted therapeutically. Epigenetic changes further modulate these pathways, enabling GBM cells to adapt to environmental stresses [25,26]. Cellular metabolism also supports DNA damage response (DDR) mechanisms by providing the necessary cellular building blocks for PTM [27]. ATP citrate lyase (ACLY) and pyruvate dehydrogenase (PDH) produce acetyl CoA (AcCoA) through glucose oxidation and undergo nuclear localization to engage in acetylation near double-stand breaks (DSBs) and promote the DDR mechanism of homologous recombination [28]. Studies also show that AcCoA and methyl groups translocate to the nucleus under DNA stress conditions to participate in DDR, playing a critical role in maintaining PTMs [28,29]. This suggests that metabolic alterations support PTM response and can be used as a predictor for patient survival and treatment efficacy. Therefore, identifying metabolite drivers of PTM and targeting the interconnected metabolic and epigenetic mechanisms holds promise for disrupting tumor survival strategies, which is critical for improving GBM patient outcomes.

In recent years, antibody–drug conjugates (ADCs) have emerged as a promising class of chemotherapeutics under clinical development for a range of solid tumors, including GBM [30,31,32,33]. ADCs consist of antibodies conjugated to therapeutic agents via chemical linkers, allowing precise delivery to specific targets with enhanced efficacy, selectivity, and potentially reduced toxicity compared to conventional treatments. Sacituzumab govitecan (SG) is an example of an ADC, combining a humanized anti-Trop-2 monoclonal antibody (hRS7) with SN-38, the active metabolite of irinotecan, as its cytotoxic payload. Irinotecan, a topoisomerase I inhibitor, serves as a prodrug for SN-38, which is primarily converted to its active form in the liver [34,35,36]. However, a substantial portion of SN-38 is further metabolized into an inactive form, SN-38 glucuronide (SN-38G), by the enzyme UDP glucuronosyltransferase UGT1A1, a process associated with gastrointestinal toxicity in irinotecan therapy [37,38]. We previously found that the utilization of the antibody–drug conjugate, SG, effectively delivered SN-38 to tumors in vivo without significant production of the toxic by-product SN-38G [30,32,39,40]. However, GBM is a biologically complex disease that requires multifaceted therapies to address both the inter- and intra-patient tumor variability. A synergistic drug combination is also advantageous to reduce the dosage to a minimum, thereby reducing potential side effects [41,42,43,44]. In this study, we employ high-throughput drug screening, metabolomics, and metabolic flux analysis on whole cells and sub-cellular nuclear compartments across a panel of GBM cell lines. For the first time, we report the metabolically driven epigenetic effects of the synergistic combination of SN-38 and checkpoint kinase 1 inhibitor (CHK1), rabusertib, in both +MGMT and −MGMT GBM cell lines, suggesting a promising candidate for combinatorial therapy in GBM.

## 2. Results

### 2.1. High-Throughput Drug Screening Identifies the Synergistic Drug Combination SN-38 and Rabusertib

**Figure 1:**
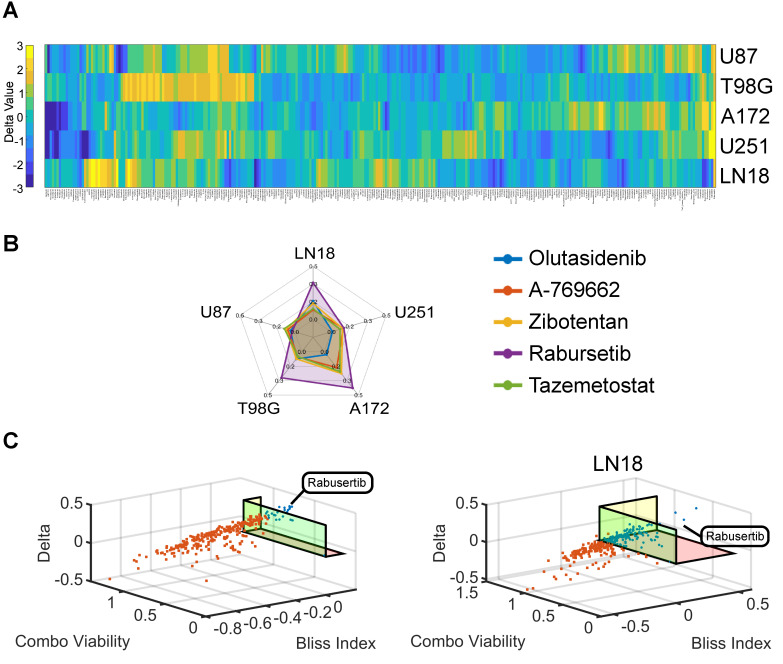
High-throughput drug screening of 292 compounds on 5 GBM cell lines (A172, LN18, T98G, U87, and U251) identifies rabusertib as a synergistic combination with SN-38. (**A**) Bliss index values of all 292 drugs with SN-38 showing rabusertib as the only drug combination that is synergistic in all cell lines. Reference table with drug compound names can be found in Supplementary Table S4. (**B**) Bliss index values of the top 5 highest scoring drug combinations with SN-38 from high-throughput drug screening in GBM cell lines. (**C**) Three-dimensional plots of Delta (Single Drug Viability–Combo Drug Viability), Bliss index, and Combo Viability values from high-throughput drug screening analysis in GBM cell lines: unmethylated MGMT cell line (LN18) and methylated MGMT cell line (U251).

Based on previous studies demonstrating the promising efficacy of SN-38 in GBM, we set out to identify drugs synergizing with SN-38 [30,39]. An unbiased high-throughput screen of a cancer drug library of 292 compounds alone and in combination with 10 nM SN-38 was conducted in five GBM cell lines (of which three were unmethylated MGMT, +MGMT, and two were methylated MGMT, −MGMT), using an ATP bioluminescence assay, as previously described [45,46,47]. The screening identified five top-hit drug combinations that synergize with SN-38 (olutasidenib, A-769662, zibotentan, tazemetostat, and rabusertib) in all five GBM cell lines, as measured by the Bliss index (Figure 1A, Supplementary Figure S1). The rabusertib and SN-38 combination had the highest Bliss index values in four out of five cell lines when compared to the other top five synergistic drug combinations (Figure 1B). Only the combination of rabusertib and SN-38 showed synergy (Bliss > 0), a delta value of >25%, and a cell viability of <80% in all five cell lines (Figure 1C, Supplementary Tables S1–S3, Supplementary Figure S1). To better understand how the MGMT status impacts the effectiveness of this combined treatment, subsequent experiments focused on U251 (−MGMT) and LN18 (+MGMT) as representatives of methylated and unmethylated MGMT model systems.

To optimize minimal drug combination doses that would result in synergistic effects, U251 and LN18 GBM cell lines were treated with a range of concentrations close to previous reports of IC50 in both drugs (SN38 IC50 ≈ 500 nM; rabusertib IC 50 ≈ 7 nM) [48,49]. Cells were treated with rabusertib (15.625–500 nM) and SN-38 (125–500 nM), resulting in several synergistic concentrations in each cell line (Supplementary Figure S2). In LN18, treatment with higher concentrations of SN-38 (500 nM) produced a synergistic effect with rabusertib at concentrations ranging from 31.25 nM to 500 nM. In U251 cells, the higher dosage of SN-38 (500 nM) also produced a synergistic effect when used with concentrations of rabusertib from 15.265 nM to 500 nM. The concentrations 500 nM SN-38 and 31.25 nM rabusertib were shown to be the lowest concentration measured that produced a Bliss index of >0.2 in both LN18 and U251 cell lines and was therefore chosen for subsequent experiments (Supplementary Figure S2).

Due to the ability of DNA-damaging agents to cause redox imbalance, which may affect the results of an ATP-based cell viability screen, the synergy of the drug combination at the optimized concentration (500 nM SN-38, 31.25 nM rabusertib) was further evaluated using the metabolomics-based PEDS algorithm. The PEDS score for whole-cell extracts of U251 and LN18 were 0.8695 and 0.8411, respectively, validating that the combination treatment of SN-38 and rabusertib synergistically affects both cellular metabolism and cell viability.

### 2.2. Combination of SN-38 and Rabusertib Alters Metabolic Pathways Related to Epigenetic Regulation

**Figure 2:**
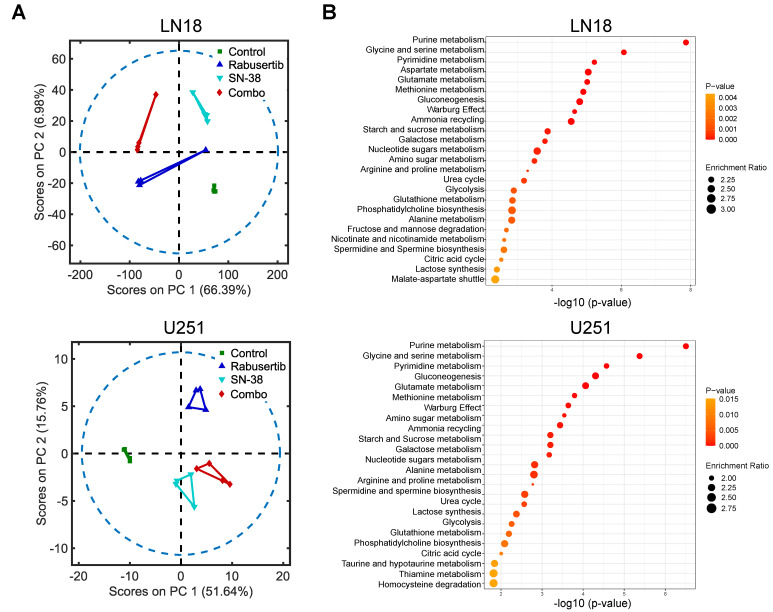
Metabolomics analysis of unlabeled whole-cell extracts shows the greatest variance between control and combination treatment groups, specifically in nucleotide, redox, and one-carbon metabolism. (**A**) Principal component analysis (PCA) of principle component (PC) 1 and PC2 and (**B**) pathway enrichment analysis obtained from MetaboAnalyst 5.0 comparing whole-cell extract metabolite features from untargeted metabolomics in control versus combination treatment groups for LN18 and U251 cell lines.

SN-38 has been shown to induce the recruitment of metabolites such as AcCoA to DSB to engage in DDR mechanisms [28]. Therefore, we hypothesized that the synergistic combination of SN-38 and rabusertib will affect metabolic pathways related to epigenetic regulation. To determine whether epigenetic-related pathways were altered by the combined SN-38 and rabusertib treatment, we conducted whole-cell metabolomics analysis. A principal component analysis (PCA) consistently revealed that the metabolic signature of the combination treatment group exhibited the greatest variance compared to the control group (LN18: PC1: 66.39%, PC2: 6.98%; U251: PC1: 51.64%, PC2: 15.76%) (Figure 2A). A subsequent pathway analysis was conducted on significant metabolomic features (Student’s *t*-test < 0.05) to determine which specific pathways were the main drivers. For both cell lines, the pathways that showed the greatest number of significant hits when comparing the control and combination groups are nucleotide metabolism and epigenetic pathways (glycine, serine, and methionine metabolism) (Figure 2B). In the comparison between the control and SN-38 treatment groups, the greatest number of significant hits in both cell lines were related to one-carbon metabolism (Supplementary Figure S3).

**Figure 3:**
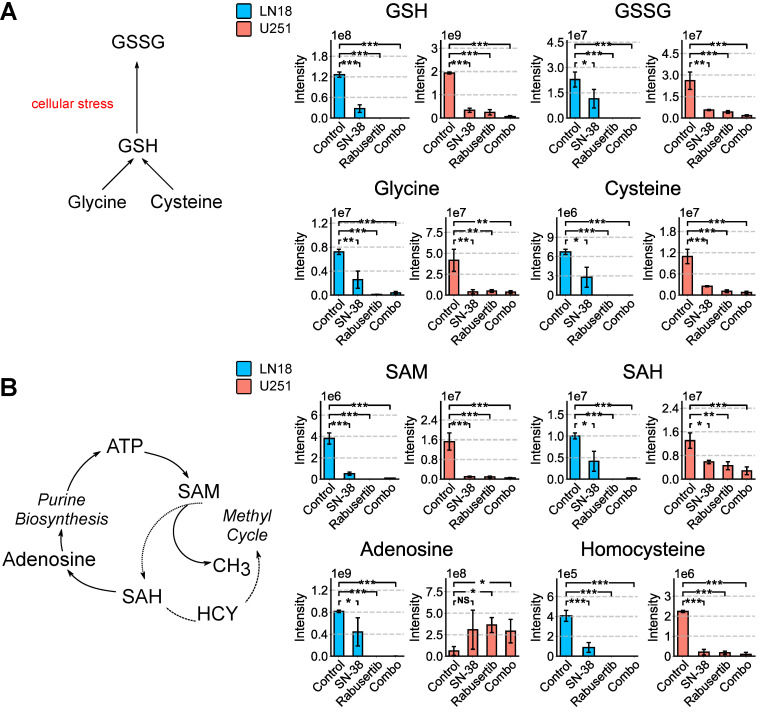
SN-38 and rabusertib treatment significantly downregulate glutathione-cycle intermediates. Total pooled intensities of (**A**) glutathione-cycle intermediates and (**B**) purine- and methyl-cycle intermediates SAM and SAH measured by LC-MS in whole-cell extracts of LN18 and U251 cells (* = *p* < 0.05; ** = *p* < 0.005; *** = *p* < 0.001; NS, *p* > 0.05). Abbreviations: GSSG, oxidized glutathione; GSH, glutathione; ATP, adenosine triphosphate; SAM, S-adenosyl methionine; SAH, S-adenosyl homocysteine; HCY, homocysteine.

In the glutathione cycle, glutathione (GSH), a major contributor to redox state modulation, was also significantly downregulated (LN18: *p* = 1.00 × 10^−3^; U251: *p* = 2.73 × 10^−9^) along with its component parts cysteine, cysteinylglycine, and glycine in the combination treatment (U251: *p* = 0.0001; *p* = 0.0001; *p* = 0.002; LN18: *p* = 1.34 × 10^−7^; *p* = 1.02 × 10^−6^; *p* = 3.61 × 10^−7^) (Figure 3A and Supplementary Figure S4). In addition, the one-carbon-cycle intermediates S-adenosyl methionine (SAM), S-adenosylhomocysteine (SAH), and homocysteine, which generate methyl groups used for methylation, were also significantly downregulated (U251: *p* = 0.01; *p* = 8.84 × 10^−4^; *p* = 5.31 × 10^−8^; LN18: *p* = 3.26 × 10^−4^; *p* = 3.121 × 10^−7^; *p* = 1.48 × 10^−5^) (Figure 3B and Supplementary Figure S5). In both cell lines, there is a reduction in one-carbon-cycle intermediates; however, in U251 cells, no significant difference is observed in purine salvage metabolites, such as xanthine and hypoxanthine, when comparing the control and combination treatments. This suggests that a high level of DNA damage repair mechanisms is occurring in the +MGMT U251 cells (Figure 3 and Supplementary Figure S5). Notably, adenosine is significantly increased (*p* < 0.05) in U251 cells, whereas homocysteine is significantly decreased (Figure 3B). This accumulation of adenosine suggests that the purine salvage pathway intermediates are not being used for repair and may instead be indicative of cell death (Figure 3B).

### 2.3. SN-38 and Rabusertib Synergistically Alter Nuclear Metabolism

Significant metabolic remodeling, including nucleotide metabolism, glutathione metabolism, and one-carbon metabolism, was observed in the whole-cell extracts of GBM cells treated with combined SN-38 and rabusertib. These pathways also localize, to some extent, in the nucleus, so we investigated the treatment-induced metabolic modulation occurring specifically in the nucleus. While the mechanism behind nuclear metabolite translocation remains incompletely understood, it is generally accepted that nuclear metabolite compartmentalization occurs within cells [50,51]. To explore this further, we optimized a nuclear isolation method and analyzed nuclear extracts from treated GBM cells (Supplementary Figure S6).

**Figure 4:**
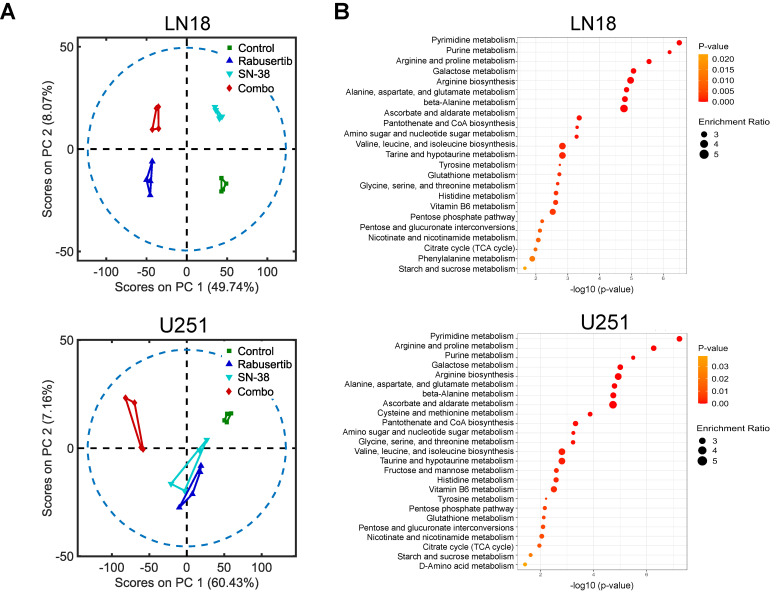
Metabolomics analysis of unlabeled nuclear extracts shows the greatest variance between control and combination treatment groups, specifically in nucleotide, redox, and one-carbon metabolism. (**A**) Principal component analysis (PCA) of principle component (PC) 1 and PC2 and (**B**) pathway enrichment analysis obtained from MetaboAnalyst 5.0 comparing significant (Student’s *t*-test, *p* < 0.05) whole-cell extract metabolite features from untargeted metabolomics in control versus combination treatment groups for LN18 and U251 cell lines.

**Figure 5:**
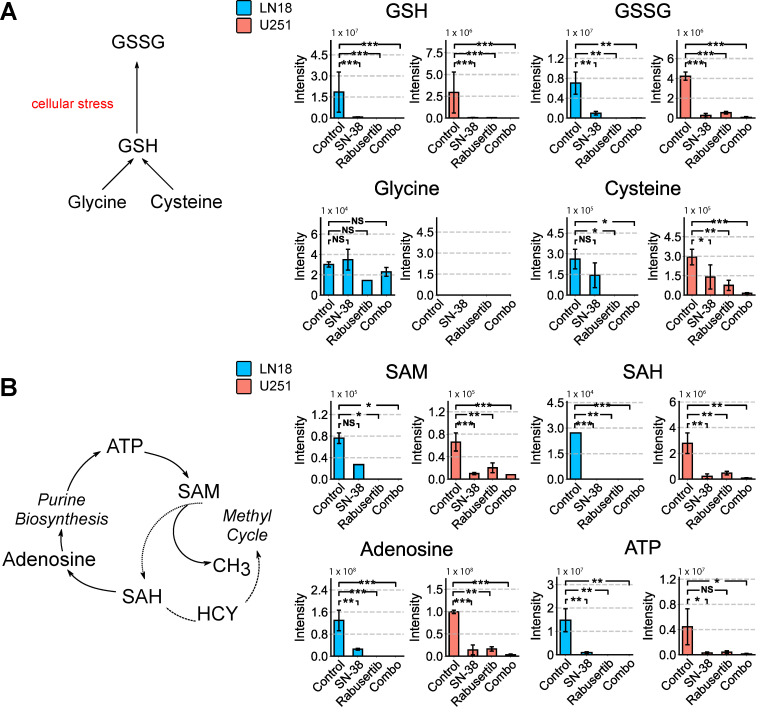
Nuclear localized metabolism shows alterations in purine, one-carbon, and redox metabolism. Total pooled intensity of nuclear (**A**) one-carbon and (**B**) purine synthesis metabolites shows significant global depletion with combination treatment in both LN18 and U251 cell lines. (* = *p* < 0.05; ** = *p* < 0.005; *** = *p* < 0.001; NS, *p* > 0.05) Abbreviations: AMP, adenosine monophosphate; ATP, adenosine triphosphate; IMP, inosine monophosphate; GMP guanosine monophosphate; SAH, S-adenosyl homocysteine; SAM, S-adenosyl methionine.

To investigate the overall synergistic effect of SN-38 and rabusertib on nuclear metabolism, we calculated the PEDS score using all detected metabolites from the isolated nuclear fraction. PEDS scores of 1.25 and 1.02 in U251 and LN18 cells, respectively, indicate higher levels of synergy compared to those observed in whole-cell extracts, suggesting that nuclear-compartmentalized metabolites significantly contribute to the synergistic effect of this combination. The PCA analysis revealed distinct clustering between treatment groups, with the control and combination groups showing the greatest difference in variance on principal component 1 (LN18: 49.74%; U251: 60.43%) compared to the other groups (Figure 4A). The pathway enrichment analysis of the comparison between the control and SN-38 groups shows that the most significant number of hits are related to galactose metabolism in LN18 cells and tryptophan metabolism in U251 cells (Supplementary Figure S7). The comparison of the control and combination groups confirmed that purine and pyrimidine metabolism was most significantly altered in the nuclei, followed by redox metabolism (arginine and proline biosynthesis) (Figure 4B). Interestingly, the one-carbon metabolism pathway was not significantly altered, possibly due to its localization in the mitochondrial and cytosolic fractions (Figure 4B). Glutathione generation was greatly decreased, as evidenced by the significant reduction in GSH-cycle intermediates following combinatorial treatment (Figure 5A and Supplementary Figure S8). In LN18 cells treated with rabusertib, GSH and GSSG levels were either very low or undetectable (Figure 5A). Moreover, a Western blot analysis of DSB marker ɣ-Histone 2A revealed an accumulation of DSB following SN-38 treatment, both alone and in combination with rabusertib in LN18 cells (Supplementary Figure S9). In U251 cells, the combination with rabusertib induced a synergistic increase in DSB, despite S-phase arrest observed in rabusertib-treated groups, as indicated by the accumulation of the S-phase marker CDK2 (Supplementary Figure S9). These findings highlight the heterogeneity in the response of GBM cell lines to the combinatorial treatment. Additionally, all purine salvage metabolites decreased precipitously (*p* < 0.05) following combination treatment, different to our observations for the whole-cell extracts (Figure 5B and Supplementary Figure S10). When examining the intersection of one-carbon-cycle and purine metabolism, adenosine did not accumulate in the nuclei as it did in the whole-cell fraction (Figure 5B).

### 2.4. Epigenetic Metabolites 5-Methyl-Cytosine, Acetyl-Lysine, and Trimethyl-Lysine Decrease with Combination Treatment of SN-38 and Rabusertib

We hypothesized that the combined effects of SN-38 and rabusertib would alter pathways related to epigenetic regulation. Indeed, we observed that in both whole-cell and nuclear extracts, one-carbon metabolites and purine metabolites that feed one-carbon metabolism were significantly downregulated following combination treatment. Therefore, stable isotope incorporation was employed to further elucidate the compartmentalization of nucleotide metabolism, one-carbon metabolism, and redox cofactors and to examine the generation of the epigenetic metabolites 5-methyl-cytosine, acetyl-lysine, and trimethyl-lysine following combination drug treatment. [U-^13^C_6_] glucose was utilized due to glucose incorporation into purines through the pentose phosphate pathway. Additionally, glucose oxidation is used to generate available AcCoA, which has been directly linked to the ability of the cell to undergo acetylation.

**Figure 6:**
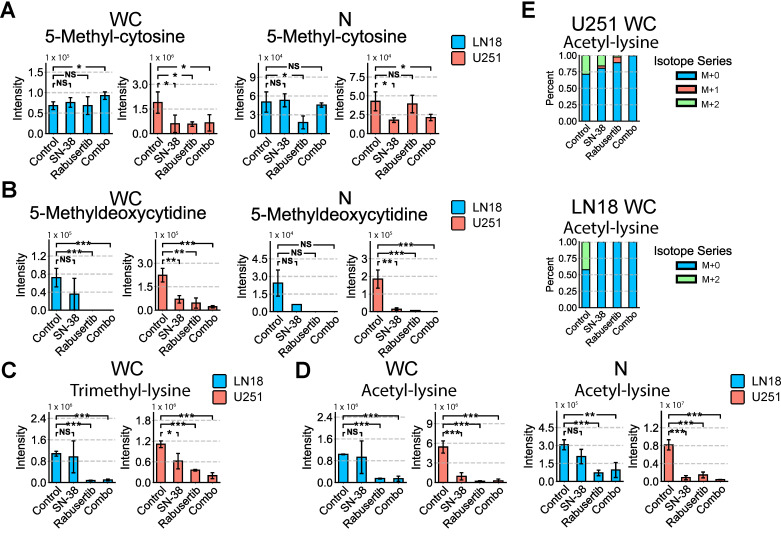
Combination drug treatment of SN-38 and rabusertib induces significant changes in epigenetic metabolism. Total pool of epigenetic metabolites (**A**) 5-methyl-cytosine (5mC) and (**B**) 5-methyldeoxycytosine (5mdC) detected in LN18 and U251 whole-cell and nuclear fractions after 24 h of treatment. (**C**) Total pools of trimethyl-lysine. (**D**) Total pooled intensity of acetyl-lysine and percent isotopic label incorporation from [U-^13^C_6_] glucose. (**E**) [U-^13^C_6_] glucose percent label incorporation to acetyl-lysine in LN18 and U251. (* = *p* < 0.05; ** = *p* < 0.005; *** = *p* < 0.001; NS, *p* > 0.05) Abbreviations: WC, whole cell; N, nuclear.

In U251 cells, 5-methyl-cytosine (5mC) decreased significantly in the combination treatment, whereas it remained unaltered in LN18 cells (Figure 6A). Interestingly, the only significant change in 5mC in LN18 cells was in the comparison of nuclear 5mC in control versus rabusertib, which was not significantly changed in U251 cells (Figure 6A). 5-methyldeoxycytidine (5mdC), an epigenetic metabolite involved in global DNA methylation through the methylation of cytosine–guanine dinucleotides, was undetectable in both whole-cell and nuclear fractions of LN18 cells after rabusertib and combination treatment. In contrast, a decreasing trend in 5mdC levels was observed in U251 whole-cell extracts (Figure 6B). ACLY and PDH, enzymes that localize to the nucleus as part of the DDR mechanism to generate AcCoA for acetylation, showed decreased expression in the isolated nuclear fraction following combination treatment in both cell lines (Supplementary Figure S11A). Additionally, PDH enzyme expression decreased with combination treatment in LN18 (Supplementary Figure S11A). Despite these changes, the histone acetyl transferase (HAT) activity in both U251 and LN18 cell lines showed a marked increase after 24 h of combinatorial treatment compared to the control (Supplementary Figure S11B).

The methylation of CpG islands is associated with gene silencing whereas lysine acetylation and methylation are associated with translation and chromatin remodeling. Lysine residues on histones can be methylated, demethylated, or trimethylated. Given that lysine residues on histones and other proteins can also be acetylated, we focused on analyzing lysine acetylation and methylation in whole-cell fractions. To assess the contribution of glucose oxidation in hypermethylation, trimethyl-lysine (3meK) isotope tracers were assessed, following [U-^13^C_6_] glucose incorporation. Interestingly, only unlabeled 3meK was detected from our analysis, and its levels significantly decreased following combination drug treatment in both cell lines (Figure 6C). The total pool of AcK also decreases with combination treatment; in fact, AcK M+2 decreases significantly with combination drug treatment (Figure 6D). Since glycolysis is a main contributor of acetyl groups needed for acetylation, the decrease in AcK M+2 coupled with the decrease in the total pool of AcK detected suggests that the combination of SN-38 and rabusertib decreases global acetylation (Figure 6E). Because acetylation is directly implicated in gene expression, SN-38 and rabusertib therefore alter global gene expression in GBM cells regardless of their MGMT status.

## 3. Discussion

GBM is a challenging cancer to treat due to several factors, including MGMT methylation status, ineffective penetration of treatment through the BBB, and the ability of GBM to evade cell death through DDR mechanisms. While traditional GBM treatments aim to induce DNA damage, alterations in these mechanisms often lead to cell-cycle arrest instead of cell death, which significantly impacts treatment efficacy. This study investigated a novel drug combination of the CHK1 inhibitor rabusertib and the DNA-damaging agent SN-38, which is the active metabolite of the FDA-approved ADC, Sacituzumab govitecan (SG) [39]. Previous studies demonstrate the ability of SN-38 and rabusertib to penetrate the BBB, making this combination clinically relevant for GBM therapy [30,39,52]. In addition to the observed synergistic cytotoxic effects of the combination treatment, SN-38 and rabusertib also synergistically altered redox metabolism and pathways involved in epigenetic modulation, both at the cellular and nuclear levels. This was evidenced by a higher PEDS score in the nuclear extracts of both cell lines when compared to the whole-cell PEDS score. Additionally, comparing pathway enrichment analysis of control versus SN-38 and control versus combo from the whole-cell lysates produced very similar results, detailing most cellular metabolic remodeling following either single SN-38 or combination treatment occurred in one-carbon metabolites. However, the pathway enrichment analysis of control/SN-38 nuclear lysates showed a vastly different metabolic landscape, with nuclear galactose remodeling in one cell line (LN18) and tryptophan alterations in the other (U251); both pathways have been implicated in GBM survival [53,54]. In comparison, the addition of rabusertib to SN-38 treatment downregulated nucleotide and redox metabolites in nuclear fractions from both cell lines, suggesting that the combination treatment reduces GBM cell survival by targeting these pathways. Given that the focus of this study was on nucleotide, redox, and one-carbon pathways, which can be found in both nuclear and cytosolic cellular compartments, subcellular fractionation techniques were critical in isolating the effects directly related to epigenetic modulation in the nucleus. These findings underscore the importance of subcellular fractionation in metabolomics analysis.

The downregulation of glutathione (GSH)-cycle intermediates in the nucleus indicates that the combination treatment induces substantial oxidative stress. Previous studies have shown that nuclear-localized reactive oxygen species (ROS) can bypass cell cycle checkpoints, thus altering cell cycle progression and gene expression [55]. Rabusertib, a CHK1 inhibitor, is known to impact the cell cycle, and our data showed very low or undetectable levels of GSH and GSSG in rabusertib-treated LN18 cells. However, it remains unclear whether the absence of GSH and GSSG in these cells affects the efficacy of rabusertib treatment.

The combination of SN-38 and rabusertib also resulted in significant epigenetic changes, likely driven by the intense cellular stress induced by the treatment, as well as prior studies emphasizing ACLY recruitment to sites of double-strand breaks (DSBs) to facilitate DDR mechanisms [28]. The localization of PDH and ACLY to the nucleus and methyl-cycle intermediates, SAM and SAH, decreased with combination treatment. The downregulation of ACLY and PDH, major contributors to histone acetylation, is indicative of a decrease in chromatin remodeling and therefore a decrease in the response of DDR mechanisms. In agreement with these findings, the epigenetic metabolites 5mC, AcK, and 3meK all significantly decreased with the combination treatment. The notable exception was 5mC in LN18 cells, which did not follow the same trend. Despite an increase in histone acetyltransferase activity at 24 h, the total pool of AcK significantly decreased in both cell lines, suggesting that the reduction in AcK was due to insufficient AcCoA rather than a lack of HAT activity. Similarly, total pools of 3meK and 5mC also decreased, reinforcing the notion that the combination treatment induces cellular stress, limiting downstream acetylation and methylation and impairing DDR processes.

Our initial drug screening results revealed rabusertib, a CHK1 inhibitor, to be highly synergistic with SN-38. Although high-throughput drug screening provides an extensive overview of drug vulnerabilities, it is limited by the number of drugs included in the library. For this study, we prioritized testing a wide range of drugs with minimal overlap in their targets to broaden our scope of understanding. Future studies may explore combinations of rabusertib with other CHK1 inhibitors to optimize responses in MGMT knockout GBM models. GBM is a heterogenous cancer containing multiple cell types, and one such subset is GBM stem cells (GSCs). The infiltration of self-renewing GBM stem cells (GSCs) to neighboring brain tissue also contributes to the aggressive and complex nature of GBM treatment, making it a prominent therapeutic target [56]. Therefore, subsequent experiments may be conducted to assess the response of GSCs to this combination treatment to understand its effects on GBM recurrence. Regardless of these limitations, MGMT status remains a major determinant of GBM treatment due to its ability to mitigate the effects of TMZ treatment. By utilizing two GBM cell lines with distinct MGMT statuses, we tested our hypothesis that the combination of SN-38 and rabusertib is unaffected by MGMT status and induces epigenetic modifications. We found that, indeed, the combination of SN-38 and rabusertib overwhelms DDR mechanisms, leading to cell death and a significant decrease in redox, purine, and one-carbon-cycle metabolites, specifically localized in the nucleus.

While GBM remains a highly variable disease, further investigation into other common genetic and epigenetic modifications is necessary. Nonetheless, MGMT continues to be a significant barrier to GBM treatment, and our findings suggest that the combination of SN-38 and rabusertib holds promise as a synergistic therapy for GBM patients, regardless of their MGMT methylation status. Future studies will focus on validating these results in animal models to assess the efficacy and safety of this combination in vivo.

## 4. Materials and Methods

### 4.1. Cell Culture

Glioblastoma cell lines (U251, LN18, A172, U87MG, T98G) were obtained from the American Type Tissue Collection (ATCC) and cultured in DMEM (Gibco, Waltham, MA, USA) supplemented with 2 mM glutamine (Gibco, Waltham, MA, USA), and either 10% characterized FBS (Cytiva, Marlborough, MA, USA), or 10% dialyzed FBS (Cytiva, Marlborough, MA, USA) for isotope-labeling experiments. Prior to starting the labeling experiments, cells were passaged twice in medium containing dialyzed FBS. Cells were maintained at 70% confluence during passaging.

### 4.2. Drug Screening for Identification of Novel Synergistic Drug Combinations

Glioblastoma cell lines were screened using a customized compound library of 292 drugs from Med Chem Express (Monmouth Junction, NJ, USA) alone and in combination with SN-38 (Med Chem Express, Monmouth Junction, NJ, USA), as previously reported [46,47,57]. All drugs were prepared in a stock solution of DMSO (Fisher Scientific, Waltham, MA, USA) and then further diluted with PBS to reach a final treatment concentration of 0.1% DMSO. Glioblastoma cell lines were seeded in 384-well polystyrene plates for high-throughput screening experiments for 12 h prior to drug treatments. After 24 h of drug treatment, cell viability data were collected by utilizing the Promega Cell Titer Glo 2.0 (Madison, WI, USA) kit as per the manufacturer’s instructions and measured using the Tecan Spark microplate reader (Männedorf, Switzerland) coupled with Magellan Data Analysis software (Männedorf, Switzerland, https://lifesciences.tecan.com/software-magellan, accessed on 10 December 2024).

Top hit combinations were determined by an in-house MATLAB script utilizing normalized cell viability measured from luminescence values. Bliss index calculations were conducted as a measurement of synergy [58,59]. Drug screening results were assessed using an in-house script to calculate the synergistic effect (>0) using the Bliss index and Delta: the difference in cell viability of single and combination treatments, and the cell viability of the combination treatment. The principal component analysis (PCA)-based Euclidean distance synergy quantification (PEDS) algorithm was utilized to assess metabolic synergy [47,57].

### 4.3. Cell Harvesting for LC-MS Analysis

For the labeling experiments, DMEM medium was prepared by supplementing with 10% dialyzed FBS and glucose was completely replaced with [U-^13^C_6_] glucose. The regular medium was switched to labeled medium at the time of treatment. After 24 h of treatment, the cells were harvested and whole-cell pellets were either extracted using dual-phase extraction (as previously described [60,61]) or fractionated to obtain the nuclear subcellular fraction and then extracted.

### 4.4. Cell Fractionation: Purification of Nuclear Fraction

To assess the treatment-induced metabolic changes in the nucleus independently from their extra-nuclear fraction, a nuclear isolation method was optimized. Harvested cell pellets were resuspended in 1 mL of KPBs and homogenized with 15–20 strokes through a syringe needle. The homogenates were centrifuged at 200× *g* for 5 min and the supernatant was transferred and further centrifuged at 800× *g* for 10 min to separate the nuclear and cytosolic fractions. The nuclear pellets were washed twice with ice-cold KPBS to limit cytosolic contamination [50]. The nuclear fraction was snap-frozen in liquid nitrogen and stored at −80 °C until LC-MS analysis. The nuclear and cytosolic fractions of four fractionated cell samples were tested by Western blot for α-tubulin to confirm the presence of the cytosolic fraction, and Histone 2A to confirm the presence of nuclei. With our optimized technique, the nuclear fraction retains its integrity while containing minimal contamination from the cytosol.

### 4.5. LC-MS Sample Preparation and Metabolic Analysis

Polar extracts (from either whole-cell or nuclear samples) were transferred to LC-MS compatible sampling vials and spiked with a mixture of labeled internal standards (IS). Quality controls (QCs) were produced for both the nuclear and whole-cell sample groups.

LC-MS analysis was conducted on the high-resolution, accurate-mass (HRAM) Orbitrap IQ-X Tribrid mass spectrometer coupled with the ultra-high-pressure liquid chromatography (UHPLC) Vanquish pump with climate controlled (4 °C) autosampler (Thermo Scientific, Waltham, MA, USA). Two columns were utilized for robust metabolomic analysis. The Waters Atlantis Premier BEH Z-HILIC column (1.7 µM, 2.1 mm × 150 mm, Waters, Milford, MA, USA) mobile phases were as follows: (A) LC-MS-grade water + 10 mM ammonium acetate, (B) 90:10 acetonitrile/LC-MS-grade water + 10 mM ammonium acetate (pH 9.2), (C) 100% acetonitrile. Chromatographic parameters were as follows: 7 min 90% mobile phase A and 10% mobile phase C, 10 min 100% mobile phase B, 18 min 90% mobile phase A and 10% mobile phase C. The Phenomenex Kinetex C18 Column (2.6 μm, 100 Å, 150 × 2.1 mm, Torrance, CA, USA) mobile phases were as follows: (A) LC-MS-grade water + 0.2% formic acid, (B) 100% methanol. Gradient separation was utilized for the Kinetex column as previously described [62]. The flow rate was set to 150 μL/min with an injection volume of 5 μL for each column. QC samples were acquired every 6 samples and signal intensity of IS was assessed to monitor system performance. Peak picking and alignment were conducted on raw files with SIEVE 2.2 software (Thermo Scientific, Waltham, MA, USA). Positive metabolite identification was matched to a library of standards by mass to charge ratio (M/Z) and retention time with an in-house MATLAB script or putatively assigned by matching M/Z to the KEGG database. PEDS analysis was conducted as previously described on unlabeled metabolomics data [47,57]. PCA analysis and loadings plots were conducted with MATLAB version 2017b with the PLS Toolbox 9.1 package. A list of significant metabolites (*p* < 0.05) measured by untargeted metabolomics was generated by Student’s *t*-test on control and combination groups for both cell lines in their nuclear and whole-cell fractions. This list of KEGG identifiers was entered into MetaboAnalyst 5.0 to visualize the metabolite enrichment set and visualize which metabolic pathways are largely affected by the combination treatment of rabusertib and SN-38 [63,64]. Pathways that had the highest amount of hits per pathway, an impact factor greater than 0.2, and a false discovery rate (FDR) less than 0.001 were considered.

### 4.6. Western Blot Analysis

ACLY antibody (sc-517267) and alpha Tubulin Antibody (DM1A) were purchased Santa Cruz Biotechnology (Dallas, TX, USA), and PDH (C54G1) and Histone 2A (D6O3A) antibodies were purchased from Cell Signaling Technology (Danvers, MA, USA). Before protein quantification, the pellets were reconstituted in RIPA buffer and sonicated. Protein quantification was analyzed by Pierce BCA assay (Thermo Scientific, Waltham, MA, USA) and a gel gradient of 4–20% (Bio-Rad, Hercules, CA, USA) was utilized for separation.

### 4.7. Global Acetylation Assay

Global histone acetylation was measured using a colorimetric-based ELISA kit (CycLex, Cellular Histone Acetylation Assay Kit CY1140, CycLex Co., Ltd., Nagano, Japan) as per the manufacturer’s instructions. Cells were seeded as previously described and treated in quadruplicate with SN-38, rabusertib, or a combination of the two for 3, 6, 12, 24, or 48 h, and the absorbance was measured at 450 nM within 10 min. Absorbance intensities were calculated as percentage fold change relative to the vehicle control of 0.1% DMSO for each time point.

## Data Availability

Raw data presented in this study are available on request from the corresponding author.

## Supplementary Materials

The following supporting information can be downloaded at: https://www.mdpi.com/article/10.3390/ijms26020474/s1.
