# Supplementary material for: Targeting Metabolic and Epigenetic Vulnerabilities in Glioblastoma with SN-38 and Rabusertib Combination Therapy
Source: Int J Mol Sci. 2025 Jan 8;26(2):474. doi: 10.3390/ijms26020474 (PMC11764980; doi:10.3390/ijms26020474)
Supplement: Supplementary file 1 [file ijms-26-00474-s001.zip › ijms-3394892-supplementary.pdf]

Supplementary Materials

Targeting Metabolic and Epigenetic Vulnerabilities in Glioblastoma with SN-38 and Rabusertib Combination Therapy

Jennifer Chiou [1,2], Valeria Impedovo [1,2], Yen Bao Huynh [1,2], Ruggiero Gorgoglione [1,2], Luiz O. F. Penalva [3,4], Alessia Lodi [1,2], Andrew J. Brenner [5] and Stefano Tiziani [1,2,6,7,*]

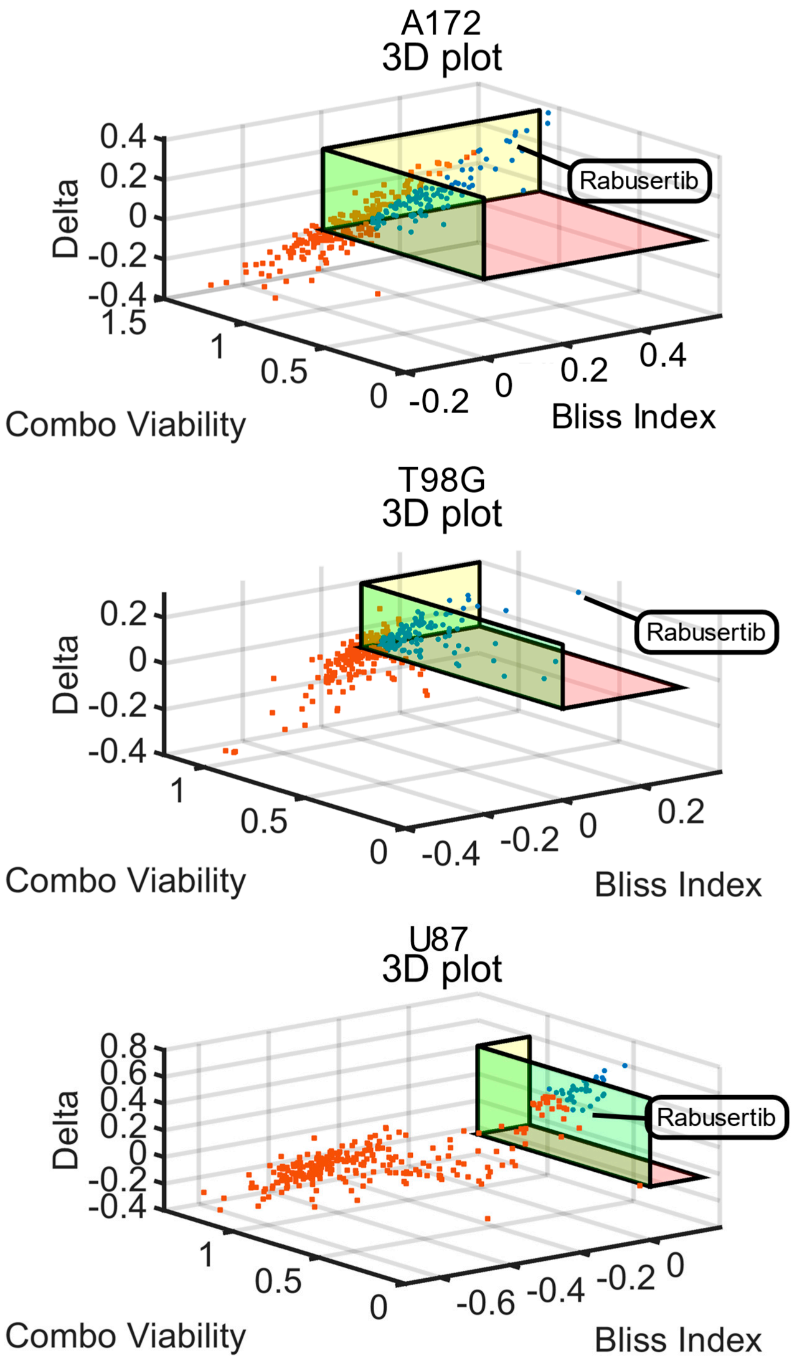

Supplementary Figure S1: High-throughput drug screening shows a distributed response of Bliss index, score, and combination viability in MGMT+ cell lines (A172, T98G) and MGMT- cell line (U87). Score values (0-1) are calculated by factoring in bliss index and the difference between cell viability of single drug treatment and combination drug treatment. Compounds with scores closer to 1 indicate effectiveness when used in combination with SN-38.

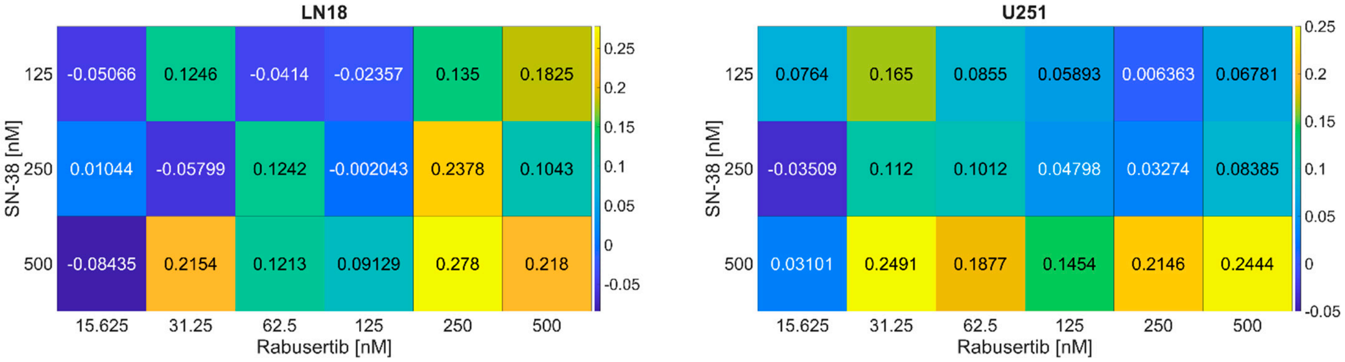

Supplementary Figure S2: Optimization of SN-38 and rabusertib concentrations show several synergistic combinations. Drug concentration optimization was conducted with 4 replicates of each condition for each cell line. Bliss index values and normalized cell viability from optimization of drug concentration for SN-38 (125 nM - 500nM) and rabusertib (15.625 nM – 500 nM) at a range of concentrations as measured by an ATP-based luminescence assay. The lowest dosage that shows similar Bliss index values between both cell lines is 500nM SN-38 and 31.25nM rabusertib.

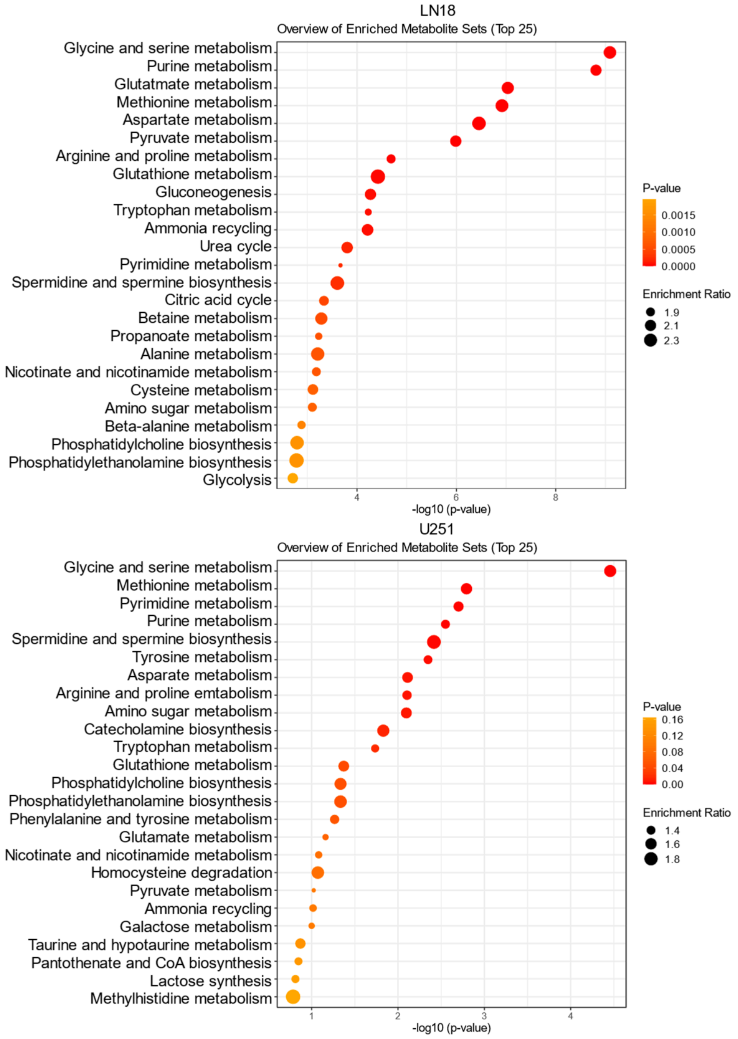

Supplementary Figure S3: Pathway enrichment analysis on LN18 and U251 whole cell lysates from the comparison of control and SN-38 treatment groups shows that the greatest number of significant features are related to one carbon metabolism.

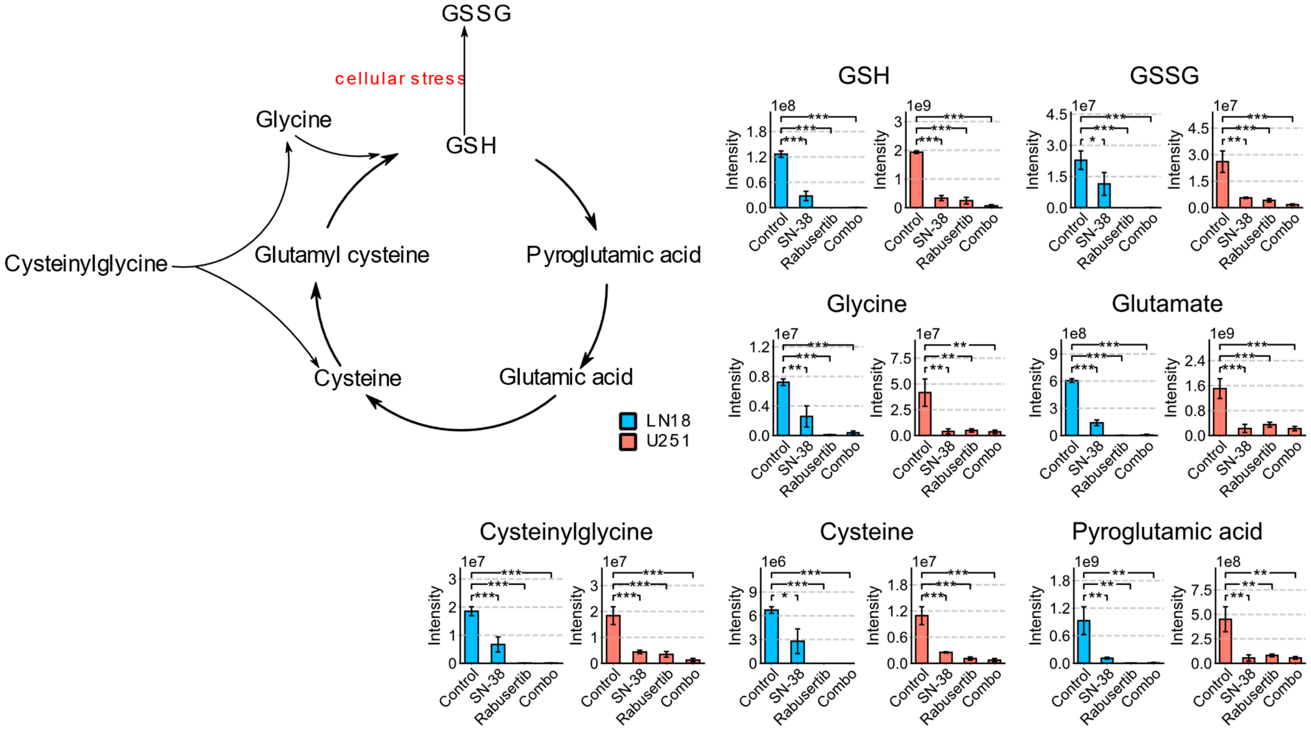

Supplementary Figure S4: Glutathione cycle intermediates are all significantly downregulated following combination treatment. Total pooled intensities of whole cell metabolites following drug treatment (control, 500 nM SN-38, 31.25 nM rabusertib, combination of SN-38 and rabusertib). (* = *p* <0.05; ** = *p* <0.005; *** = *p* <0.001; NS, *p* >0.05) Abbreviations: GSSG, oxidized glutathione; GSH, glutathione

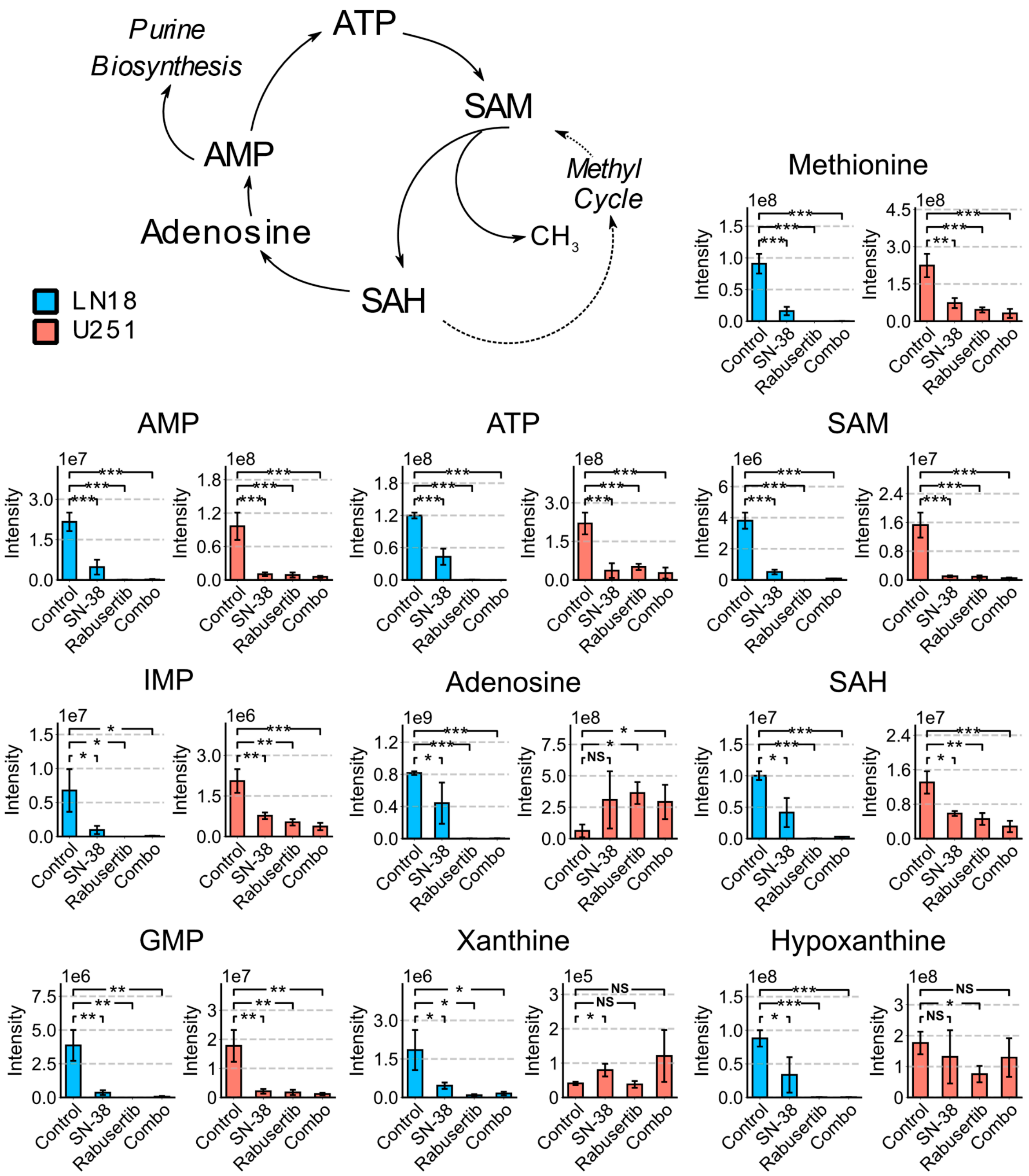

Supplementary Figure S5: Purine and one carbon pathways are interconnected through ATP and adenosine. The figure shows combination drug treatment alters one carbon metabolism in whole cell extracts of GBM cell lines LN18 and U251. Total pooled intensities of purine and one carbon cycle intermediates in LN18 and U251 cell lines show a varied downstream effect in purine salvage metabolism. (*= p <0.05; ** = p <0.005; *** = p <0.001; NS, p >0.05) Abbreviations: AMP, adenosine monophosphate; ATP, adenosine triphosphate; IMP, inosine monophosphate; GMP guanosine monophosphate; SAH, S-adenosyl homocysteine; SAM, S-adenosyl methionine

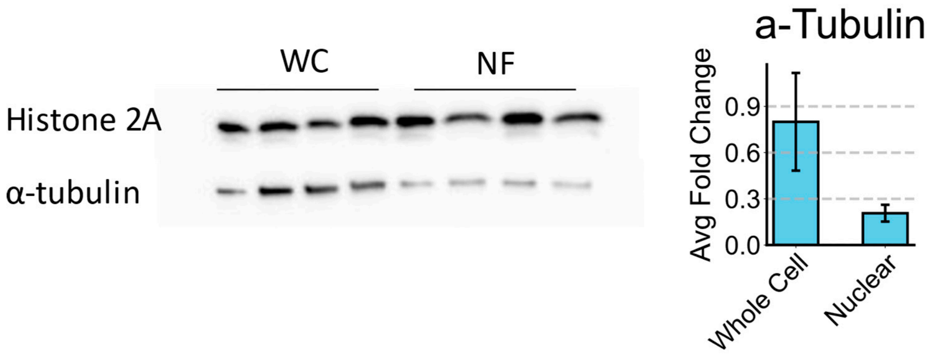

Supplementary Figure S6: Western blot (left panel) shows that following a density gradient centrifugation technique coupled with subsequent washing using KPBS produces a pure nuclear fraction that is compatible with LC-MS analysis. α-Tubulin was used as a cytosolic marker to show minimal cytosolic contamination in the isolated nuclear fraction (right panel). Histone 2A was used as a nuclear control and all fold changes values are reference to measured Histone 2A expression.

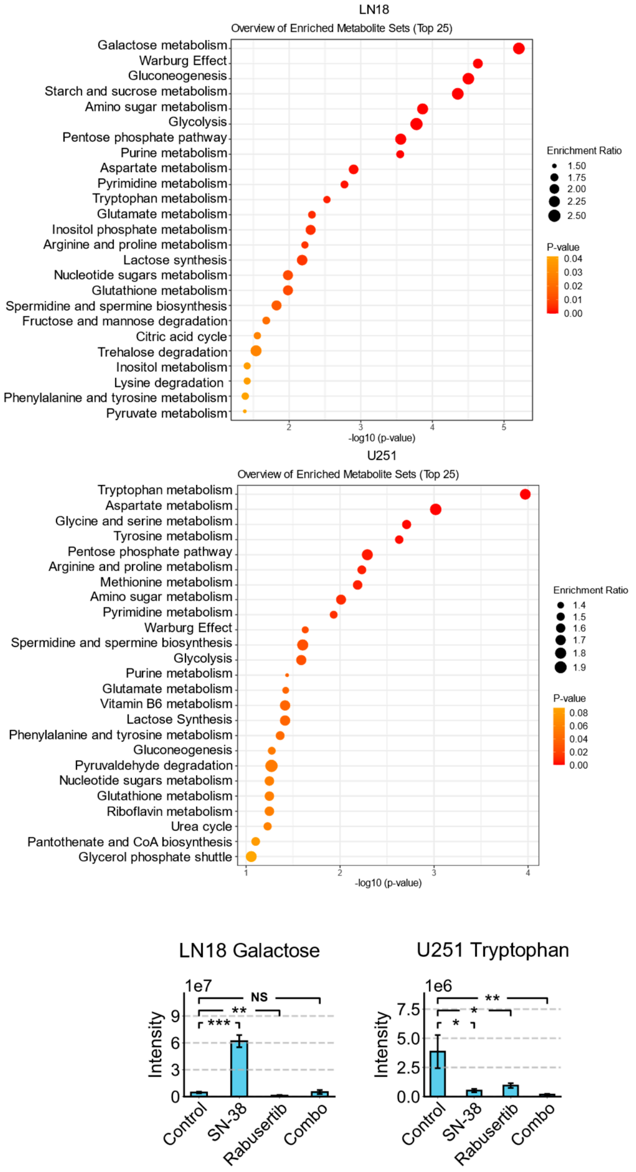

Supplementary Figure S7: Pathway enrichment analysis of LN18 and U251 nuclear lysates control and SN-38 treatment groups shows SN-38 treatment altered galactose metabolism in LN18 cells and tryptophan metabolism in U251 cells. (* = *p* <0.05; ** = *p* <0.005; *** = *p* <0.001; NS, *p* >0.05)

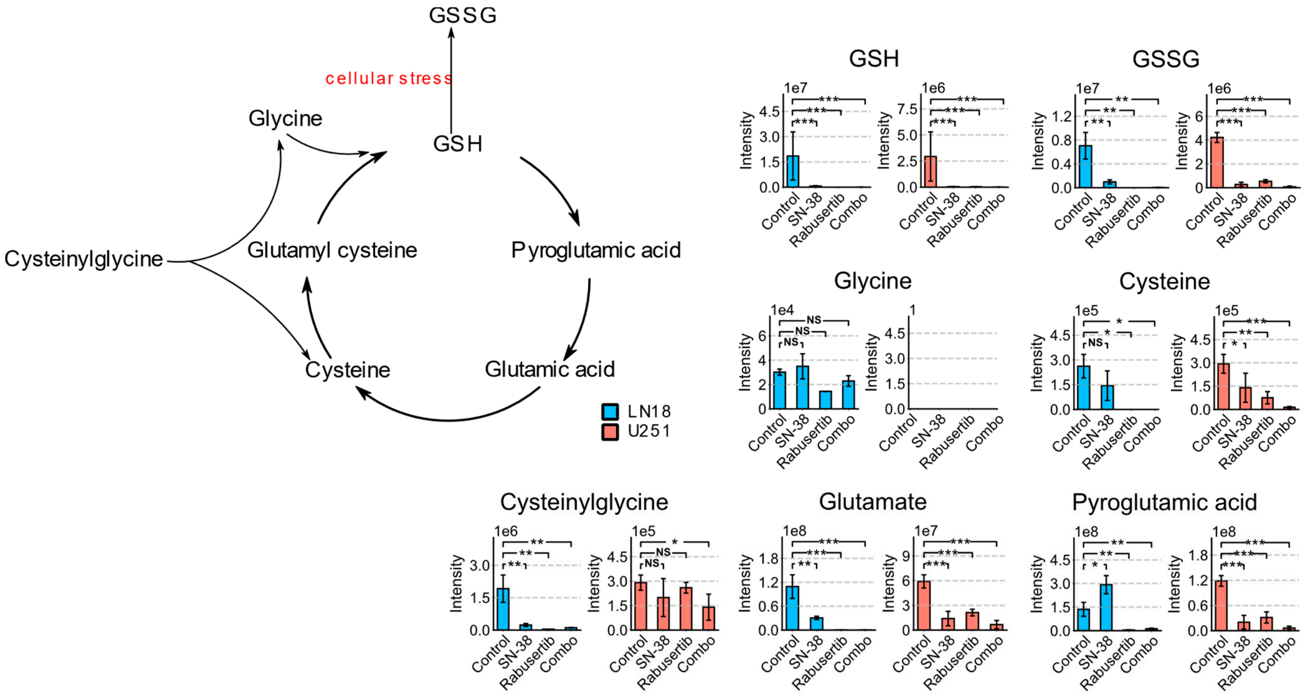

Supplementary Figure S8: Total pool of glutathione cycle metabolites from isolated nuclear fractions treated with control, SN-38, rabusertib, or the combo (SN-38 and rabusertib). Combo groups of both LN18 and U251 cell lines show widespread depletion of glutathione cycle metabolites (* = *p* <0.05; ** = *p* <0.005; *** = *p* <0.001; NS, *p* >0.05) Abbreviations: GSSG, oxidized glutathione; GSH, glutathione

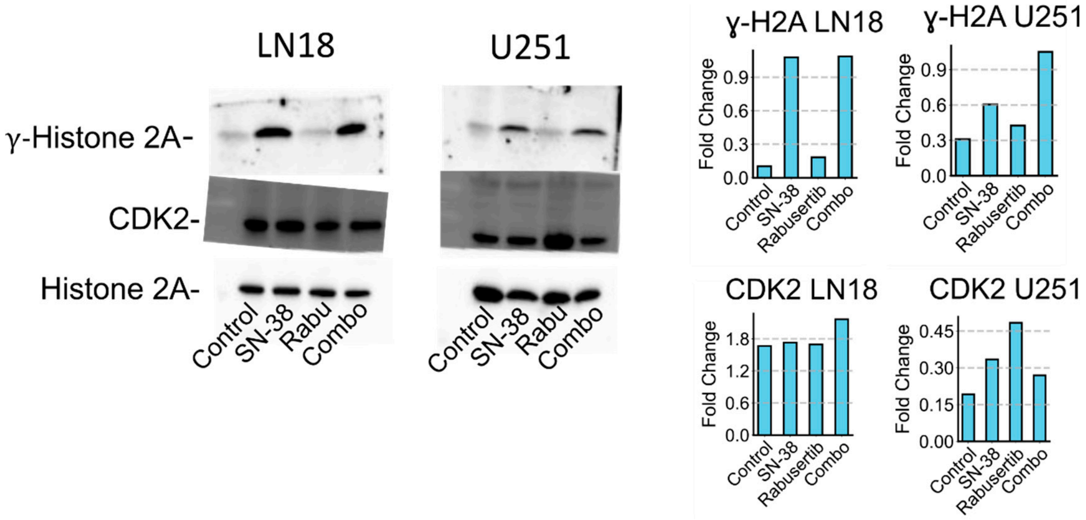

Supplementary Figure S9: Combination treatment with SN-38 and rabusertib overcome cell cycle arrest from single treatment rabusertib and induce DNA damage. Double strand break marker γ-Histone 2A, and CDK2 expression in nuclear lysates of LN18 and U251 cell lines treated with control, SN-38, rabusertib, or combination for 24 hours. In LN18 cells, western blot analysis of ɣ-Histone 2A revealed an accumulation following SN-38 treatment, both alone and in combination with rabusertib. In U251 cells, the combination with rabusertib induced a synergistic increase of ɣ-Histone 2A. All intensities were normalized and expressed relative to respective

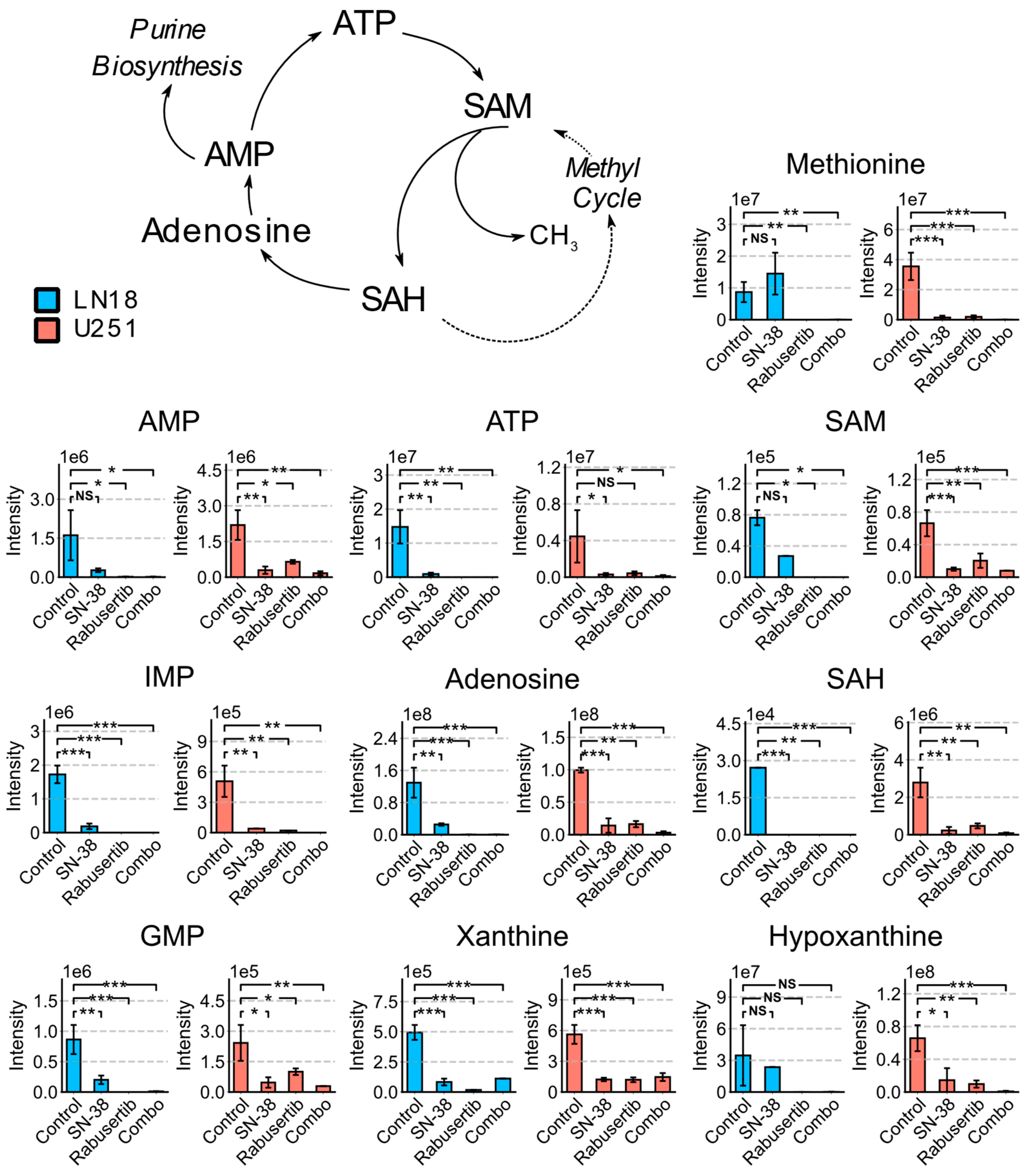

Supplementary Figure S10: Purine and one carbon pathways are interconnected through ATP and adenosine. The figure shows combination drug treatment alters one carbon metabolism in whole cell extracts of GBM cell lines LN18 and U251. Total pooled intensities of purine and one carbon cycle intermediates in LN18 and U251 cell lines shows a varied downstream effect in purine salvage metabolism. (* = p <0.05; ** = p <0.005; *** = p <0.001; NS, p >0.05) Abbreviations: AMP, adenosine monophosphate; ATP, adenosine triphosphate; IMP, inosine monophosphate; GMP guanosine monophosphate; SAH, S-adenosyl homocysteine; SAM, S-adenosyl methionine.

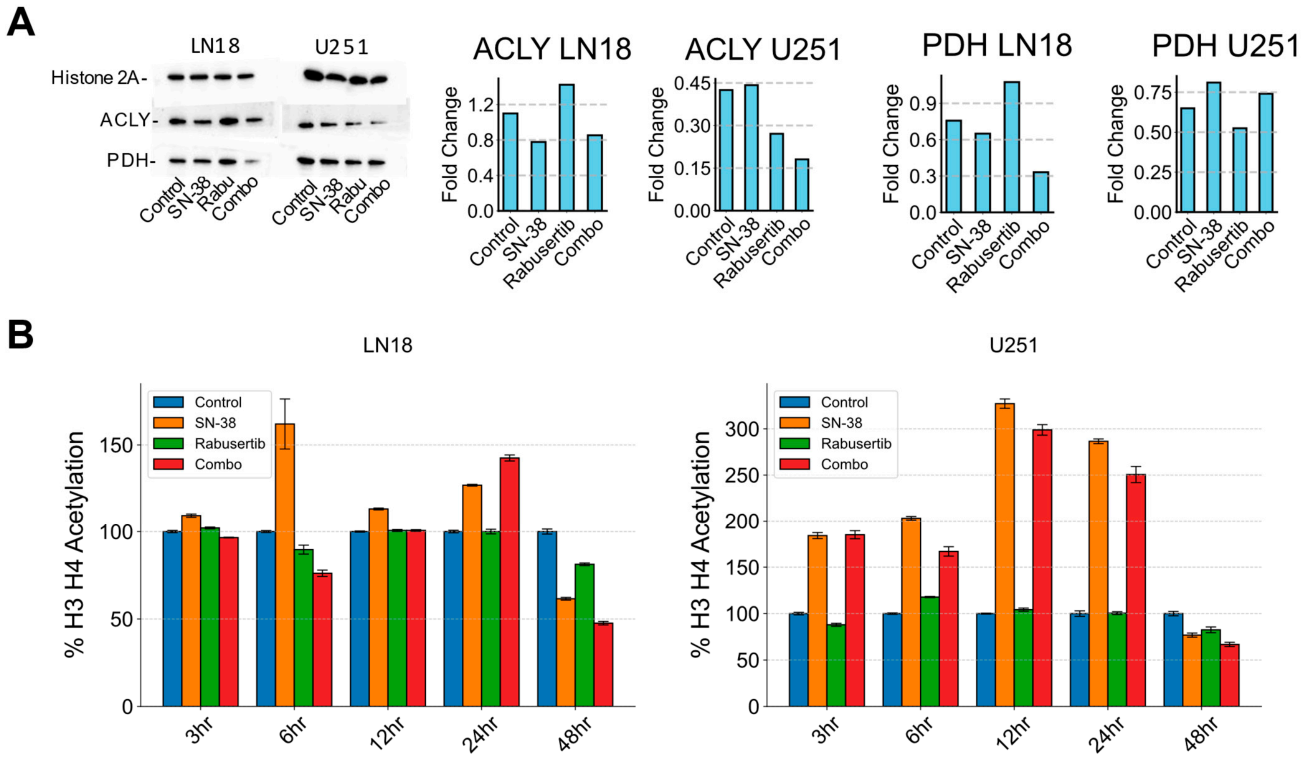

Supplementary Figure S11: Acetylation is driven by availability of metabolites to generate acetyl groups, expression of enzymes that generate acetyl groups, and activity of histone acetyl transferases (HAT) that transfer acetyl groups to histones. (A) Expression of ATP citrate lyase enzyme (ACLY) and pyruvate dehydrogenase (PDH) in nuclear extracts of LN18 and U251 cells after 24 hours of drug treatment with control, SN-38, rabusertib (Rabu), or the combination. Histone 2A was used as a nuclear control and all fold changes values are reference to measured Histone 2A expression. (B) Histone acetyl transferase (HAT) activity at 3, 6, 12, 24 hours after drug treatments (control, SN-38, rabusertib, combination) in U251 and LN18 cells. Abbreviations: ACLY, ATP citrate lyase; PDH, pyruvate hydrogenase; Rabu; rabusertib; HAT, histone acetyl transferase; H3, histone 3; H4, histone 4.

Supplementary Table S1. Top 10 drugs ranked by number of cell lines that a Bliss index of >0 following combination treatment with SN-38

Drug name	A172	LN18	T98G	U87	U251	Count
Rabusertib	0.418626	0.345088	0.300133	0.021869	0.11419	5
Olutasidenib	0.03199	0.17372	0.0831	0.037918	0.009624	5
A769662	0.182835	0.091267	0.058502	0.06479	0.109064	5
Zibotentan	0.250606	0.151375	0.080795	0.079842	0.103076	5
Tazemetostat	0.228538	0.100287	0.055761	0.094925	0.079195	5
Lomustine	0.09585	0.113566	0.079642	0.062222	-0.17498	4
KU60019	0.199288	0.232319	0.104842	8.41E-05	-0.03984	4
Trametinib	0.031711	0.143649	0.123165	-0.12932	0.000943	4
PF04217903	0.160857	0.076685	0.064869	0.012718	-0.03919	4
ND646	0.281537	0.100624	0.019331	0.09638	-0.1565	4

Supplementary Table S2. Top 10 drugs ranked by number of cell lines that have a delta value of >25% following combination treatment with SN-38

Drug name	LN18	U251	A172	T98G	U87	Count
Rabusertib	0.342803	0.411849	0.304164	0.279705	0.467128	5
Idelalisib	0.25693	0.312936	-0.13585	0.05119	0.395676	3
Olutasidenib	0.171069	0.30328	-0.08025	0.056853	0.51724	2
KU-60019	0.22959	0.25388	0.070595	0.078176	0.502061	2
Trametinib	0.141219	0.282729	-0.0789	0.095443	0.275433	2
Celecoxib	0.10589	0.250839	0.121716	-0.02959	0.458539	2
PF-04217903	0.074109	0.250828	0.046254	0.038411	0.480905	2
A-769662	0.08871	0.41265	0.076675	0.033931	0.56131	2
Raloxifene	0.080495	0.281968	0.060283	0.047617	0.44003	2
PF-4708671	0.013888	0.151727	0.309869	-0.00627	0.510551	2

Supplementary Table S3. Top 10 drugs when ranked by number of cell lines that have a cell viability <80% following combination treatment with SN-38

Drug name	LN18	U251	A172	T98G	U87	Count
Rabusertib	0.461442	0.620306	0.784798	0.507876	0.496327	5
Sepantronium	0.078267	0.018605	0.397006	0.730432	0.031165	5
Alvespimycin	0.709497	0.738255	0.75945	0.683621	0.798056	5
AZD-7762	0.363847	0.754227	0.604784	0.213134	0.620009	5
Dasatinib	0.563548	0.724861	0.766516	0.602562	0.500625	5
Torin 2	0.346011	0.612022	0.841678	0.255216	0.676962	4
Sapanisertib	0.53539	0.528332	0.815284	0.482803	0.769558	4
Obatoclax	0.473447	0.782961	1.055997	0.68267	0.378936	4
Telaglenastat	0.695118	0.703624	0.871134	0.753191	0.570836	4
Omipalisib	0.38805	0.618465	0.812294	0.369414	0.717638	4

Supplementary Table S4. Reference table with drug compound names.

Drug name	Target
Mocetinostat	Apoptosis; Autophagy; HDAC
2-Methoxyestradiol	Isocitrate Dehydrogenase (IDH)
Olutasidenib	Apoptosis; Fatty Acid Synthase (FASN)
Lomustine	Apoptosis; Autophagy; MEK
TVB-3166	c-Met/HGFR
KU-60019	PARP
Trametinib	Apoptosis; Autophagy; Bcr-Abl; CRISPR/Cas9; Microtubule/Tubulin
Celecoxib	Aromatase
PF-04217903	Others
AZD3965	Autophagy; Histone Methyltransferase
PJ34	Apoptosis; CDK
Lansoprazole	Tryptophan Hydroxylase
Nocodazole	Monocarboxylate Transporter
Megestrol acetate	Aromatase
Anastrozole	c-Met/HGFR; VEGFR
Febuxostat	Autophagy; mTOR; PI3K
BI-4924	Apoptosis; IKK; STAT
Methotrexate	Autophagy; Endogenous Metabolite
BIX-01294	Androgen Receptor
IACS-010759	Apoptosis; FGFR; VEGFR
ND-646	Autophagy; Estrogen Receptor/ERR
Danusertib	Autophagy; Bcl-2 Family; Parasite
PHA-793887	Apoptosis; Autophagy; Casein Kinase; DNA-PK; PI3K
Idelalisib	PARP
Telotristat	Hedgehog; Smo
Elesclomol	Apoptosis; CDK
BAY-8002	Apoptosis; SphK; Wnt
Serdemetan	Autophagy; Hedgehog
Exemestane	Isocitrate Dehydrogenase (IDH)
PF-3845	Bcl-2 Family
BMS-794833	Apoptosis; Autophagy; HSP
Sirtinol	TGF-β Receptor
Omipalisib	Apoptosis; Autophagy; JAK
SB 216763	Autophagy; Mitophagy; PARP
TPCA-1	IGF-1R; Insulin Receptor
Degrasyn	Apoptosis; Autophagy; DNA-PK; mTOR; PI3K
Ursolic acid	Apoptosis; HDAC
Efaproxiral	Autophagy; HDAC
GTx-007	Apoptosis; Aurora Kinase
Mitoxantrone (dihydrochloride)	Apoptosis; JAK
PD173074	Aurora Kinase
Maraviroc	Apoptosis; Autophagy; MEK

Niraparib	Autophagy; Survivin
A-769662	Apoptosis; Autophagy; Filovirus; HDAC; Mitophagy
Raloxifene (hydrochloride)	Antibiotic; Bacterial; HIV; Neurokinin Receptor
TAK-733	PKC
Obatoclax (Mesylate)	Apoptosis; Aurora Kinase; CDK
Daunorubicin (hydrochloride)	SGLT
LY294002	ROR
IM156	Antibiotic; Autophagy; Bacterial; DNA Methyltransferase; Nucleoside Antimetabolite/Analog
Rucaparib	Apoptosis; Endothelin Receptor
BMS 777607	Microtubule/Tubulin
SANT-1	Farnesyl Transferase
Galunisertib	Apoptosis; Autophagy; mTOR
SNS-032	Apoptosis; Autophagy; FKBP; mTOR
Tamoxifen (Citrate)	Free Fatty Acid Receptor; Potassium Channel
SKI II	Glutaminase
DAPT	Autophagy; PARP
Vismodegib	Apoptosis; Autophagy; Mitophagy; mTOR
PHA-665752	Autophagy; FLT3; PDGFR
Enasidenib	Autophagy; Bcr-Abl
Zileuton	Autophagy; IKK; PDK-1
AZD-5991	Aurora Kinase; Autophagy
YM-201636	Others
Bempedoic acid	Apoptosis; EGFR
PF-4708671	Apoptosis; IGF-1R; Insulin Receptor
Luminespib	Autophagy; p38 MAPK; Raf
Denifanstat	Autophagy; HSP
SB-431542	Microtubule/Tubulin; Src
FR 180204	Aurora Kinase
Momelotinib	Apoptosis; Autophagy; Bcr-Abl; Src
GOT1 inhibitor-1	DNA Methyltransferase
Olaparib	γ-secretase
SB 525334	EGFR; HDAC
Linsitinib	LXR
Letrozole	FAK; Pyk2
PI-103	Apoptosis; Autophagy; c-Met/HGFR
NU6027	Akt; Apoptosis; Autophagy
Pracinostat	Autophagy; Src
FT113	c-Kit; VEGFR
Belinostat	Apoptosis; Autophagy; Endogenous Metabolite; Microtubule/Tubulin; Reactive Oxygen Species
Dacomitinib	Apoptosis; Autophagy; DNA Alkylator/Crosslinker
Barasertib-HQPA	ATM/ATR

Belzutifan	COX
Fedratinib	Monocarboxylate Transporter
Bicalutamide	Bacterial; Phospholipase; Proton Pump
Enzastaurin	Autophagy; HIV; Progesterone Receptor
Ruxolitinib	Xanthine Oxidase
CYC-116	ADC Cytotoxin; Antifolate; Apoptosis; DNA/RNA Synthesis
TAME	Apoptosis; Mitochondrial Metabolism
Mirdametinib	Autophagy; PI3K
Luxeptinib	Apoptosis; Reactive Oxygen Species
Sepantronium (bromide)	Apoptosis; E1/E2/E3 Enzyme; MDM-2/p53
QNZ	Autophagy; FAAH
Vorinostat	Apoptosis; Autophagy; Sirtuin
Cladribine	Autophagy; GSK-3
Aprepitant	Apoptosis; Autophagy; Bcr-Abl; Deubiquitinase
Setanaxib	Reactive Oxygen Species
Sotrastaurin	Endogenous Metabolite; PKC; Topoisomerase
ABT-751	CCR; HIV
JNJ-7706621	MEK
Vatalanib (dihydrochloride)	ADC Cytotoxin; Antibiotic; Apoptosis; Autophagy; Bacterial; DNA/RNA Synthesis; Topoisomerase
Canagliflozin	AMPK
Quizartinib	c-Met/HGFR; TAM Receptor
Cintirorgon	TGF-β Receptor
KPLH1130	Apoptosis; Autophagy; Estrogen Receptor/ERR; HSP
5-Azacytidine	Amyloid-β; Apoptosis; Autophagy; Notch; γ-secretase
HM43239	Apoptosis; Autophagy; c-Met/HGFR
OSI-930	Ferroptosis; Lipoxygenase
Y-27632 (dihydrochloride)	Autophagy; Influenza Virus; PI3K; PIKfyve
Zibotentan	Fatty Acid Synthase (FASN)
TW-37	Apoptosis; ERK
Plinabulin	Others
PTC299	TGF-β Receptor
Tipifarnib	Aromatase; Autophagy
PAC-1	ATM/ATR; CDK
AZD-8055	Fatty Acid Synthase (FASN)
SN-38	Apoptosis; EGFR
Everolimus	HIF/HIF Prolyl-Hydroxylase
Volasertib	Androgen Receptor; Autophagy
GW9508	APC
Lomeguatrib	Btk; FLT3
IPN60090	NF-κB; TNF Receptor
Torin 2	Adenosine Deaminase; Apoptosis
Veliparib	Ferroptosis; NADPH Oxidase
Etoposide	Autophagy; Microtubule/Tubulin

Torkinib	Apoptosis; VEGFR
Vemurafenib	Apoptosis; Autophagy; FLT3; Ligands for Target Protein for PROTAC
Crenolanib	PDHK
AG-270	Apoptosis; FLT3
XAV-939	Bcl-2 Family
Rabusertib	Dihydroorotate Dehydrogenase; DNA/RNA Synthesis; VEGFR
Nilotinib (monohydrochloride monohydrate)	Apoptosis; Autophagy; Caspase
PIK-93	ADC Cytotoxin; Autophagy; Topoisomerase
BX795	Apoptosis; Polo-like Kinase (PLK)
WZ4002	DNA Methyltransferase
Tozasertib	Apoptosis; Autophagy; DNA-PK; mTOR
R406 (free base)	Antibiotic; Apoptosis; Autophagy; Bacterial; Mitophagy; Topoisomerase
NCT-503	Autophagy; Raf
Entinostat	Others
AEE788	PI3K; PI4K; Virus Protease
AG14361	EGFR
GSK1904529A	Apoptosis; FLT3; Syk
SGI-1776	Apoptosis; Autophagy; HDAC
Doramapimod	PARP
Fingolimod (hydrochloride)	Apoptosis; Autophagy; Pim
BIIB021	LPL Receptor; PAK
WYE-354	Apoptosis; Autophagy; mTOR
Tirbanibulin	Apoptosis; Aurora Kinase; FGFR; FLT3; PDGFR; Src; VEGFR
ENMD-2076	Fatty Acid Synthase (FASN)
SNS-314 (mesylate)	Btk; Ligands for Target Protein for PROTAC
trans-C75	Aurora Kinase
Dapagliflozin	Others
Tigecycline	ATM/ATR; Autophagy
Dasatinib	RET
Ibrutinib	Apoptosis; Bcr-Abl; FLT3; Src
GSK-3685032	DNA Methyltransferase
AMG 900	Fatty Acid Synthase (FASN)
MK-0752	Monocarboxylate Transporter
SHIN1	Others
CUDC-101	Apoptosis; Autophagy; PI3K
KU-55933	Apoptosis; PI3K
GW3965 (hydrochloride)	ADC Cytotoxin; AMPK; Antibiotic; Apoptosis; Autophagy; Bacterial; HBV; HIV; Mitophagy; Topoisomerase
Pralsetinib	Apoptosis; Autophagy; c-Fms; c-Kit; FLT3; PDGFR; VEGFR
PF-562271	mTOR
Rebastinib	Apoptosis; Mitochondrial Metabolism
SU11274	Endogenous Metabolite; GLUT; SGLT

RG108	Apoptosis; Autophagy; Bcl-2 Family; Mitophagy
MK-2206 (dihydrochloride)	Androgen Receptor
TVB-3664	Raf
Saracatinib	E1/E2/E3 Enzyme; MDM-2/p53
VB124	Autophagy; NAMPT
Motesanib (Diphosphate)	Apoptosis; Endogenous Metabolite; HIV; Nucleoside Antimetabolite/Analog
AVN-944	Autophagy; Ferroptosis; Indoleamine 2,3-Dioxygenase (IDO); RIP kinase
Iniparib	Autophagy; HMG-CoA Reductase (HMGCR)
Prinomastat	Mps1
Pictilisib	Apoptosis; Endogenous Metabolite; Microtubule/Tubulin
Siremadlin	PI3K
Buparlisib	ADC Cytotoxin; Apoptosis; Autophagy; Microtubule/Tubulin
(E)-Daporinad	Indoleamine 2,3-Dioxygenase (IDO)
Doxorubicin (hydrochloride)	Apoptosis; Drug Metabolite; Endogenous Metabolite
5-Fluorouracil	Phosphodiesterase (PDE)
Linifanib	Antibiotic; Apoptosis; Bacterial; Endogenous Metabolite; Fungal
Necrostatin-1	Autophagy; FXR
KU-0063794	PPAR
Atorvastatin	Autophagy; Casein Kinase
Devimistat	Polo-like Kinase (PLK)
AZ3146	Apoptosis; Epigenetic Reader Domain; Polo-like Kinase (PLK)
Phloretin	Influenza Virus; SGK
Docetaxel	15-PGDH
ABT-737	Apoptosis; Epigenetic Reader Domain; Histone Acetyltransferase
CH5132799	Autophagy; Bcl-2 Family
Flutamide	Aldehyde Dehydrogenase (ALDH); Interleukin Related; Pyroptosis
Paclitaxel	JAK
GDC-0879	Lactate Dehydrogenase
Epacadostat	Apoptosis; Ligands for E3 Ligase; Molecular Glues
JNJ-38877605	Microtubule/Tubulin
Bafetinib	CETP
Mycophenolate Mofetil	Autophagy; FXR
MG 149	Apoptosis; Telomerase
Anagrelide (hydrochloride)	CDK
Venetoclax	Monocarboxylate Transporter
Mycophenolic acid	Apoptosis; DNA/RNA Synthesis; Nucleoside Antimetabolite/Analog
Disulfiram	Apoptosis; Hexokinase; Mitochondrial Metabolism
GW 4064	Apoptosis; JAK
CEP-33779	Acetyl-CoA Carboxylase
GSK3787	FGFR; PDGFR; VEGFR
Galloflavin	Smo

Silmitasertib	Apoptosis; Autophagy; DNA Alkylator/Crosslinker
Lenalidomide	DNA Alkylator/Crosslinker
GSK461364	Apoptosis; Autophagy; Nucleoside Antimetabolite/Analog
Combretastatin A4	E1/E2/E3 Enzyme; MDM-2/p53
BI 2536	Androgen Receptor; Autophagy
Dalcetrapib	STAT
GSK 650394	Bcl-2 Family
Turofexorate isopropyl	Autophagy; EGFR
SW033291	Apoptosis; Ras
BIBR 1532	JAK
Crizotinib	Apoptosis; Kinesin
Temsirolimus	Apoptosis; HSP
Seliciclib	Apoptosis; MEK
Clofarabine	Apoptosis; NF-κB
Syrosingopine	Antibiotic; Apoptosis; Autophagy; Bacterial; Ferroptosis; NF-κB
Nutlin-3	Autophagy; Ferroptosis; HMG-CoA Reductase (HMGCR)
Capecitabine	Apoptosis; Monoamine Oxidase
Enzalutamide	Apoptosis; Autophagy
Lonidamine	Calcium Channel; Gutathione S-transferase; NF-κB
Homoharringtonine	Autophagy; Microtubule/Tubulin
Tofacitinib	Autophagy; Keap1-Nrf2
Gossypol (acetic acid)	Apoptosis; Raf
Firsocostat	Drug Metabolite; EGFR; IGF-1R
Gefitinib	Antibiotic; Autophagy; Bacterial; Complement System; Glucocorticoid Receptor; Mitophagy; SARS-CoV
Nintedanib	Apoptosis; DNA Methyltransferase; Nucleoside Antimetabolite/Analog
NSC 23766 (trihydrochloride)	Apoptosis; Microtubule/Tubulin
Sonidegib	Autophagy
NVP-BSK805 (dihydrochloride)	Calcium Channel; Cytochrome P450; P-glycoprotein
Temozolomide	Sirtuin
Ispinesib	Acyltransferase; Apoptosis; CMV; COX; HSV; Influenza Virus
Altretamine	Apoptosis
Alvespimycin (hydrochloride)	Autophagy; mTOR
PF-573228	Arginase
Adavosertib	GSK-3
Selumetinib	PDGFR; VEGFR
AZ7550 Mesylate	VEGFR
Triptolide	Autophagy; Bcr-Abl; Src
Dexamethasone	Raf
Sulfasalazine	Apoptosis; Ferroptosis; NF-κB
Decitabine	Lactate Dehydrogenase

Fluvastatin (sodium)	Others
Vincristine (sulfate)	Apoptosis; CDK
Isatin	Akt; AMPK; Autophagy
Genipin	Apoptosis
Apocynin	Checkpoint Kinase (Chk)
Verapamil (hydrochloride)	Autophagy; Glucocorticoid Receptor; NO Synthase; Progesterone Receptor
Ethacrynic acid	Apoptosis; Aurora Kinase; Autophagy
Selisistat	Indoleamine 2,3-Dioxygenase (IDO)
Cabazitaxel	c-Kit; FGFR; PDGFR; RET; VEGFR
Xanthohumol	Autophagy; Glutaminase
Ezetimibe	c-Kit; PDGFR; VEGFR
Azathioprine	NAMPT
AZ 628	Acetyl-CoA Carboxylase
Sapanisertib	AMPK; ATP Citrate Lyase
PH-797804	Apoptosis; c-Fms; c-Kit; VEGFR
Topotecan (Hydrochloride)	SGLT
Numidargistat (dihydrochloride)	c-Met/HGFR
GSK-690693	Apoptosis; FAK
AR-A014418	Cathepsin
SecinH3	Apoptosis; PARP
Axitinib	Apoptosis; Autophagy; PKC
AZD-7762	PARP; β-catenin
Tivozanib	Influenza Virus; PARP
Mifepristone	ALK; Autophagy; c-Met/HGFR; ROS
Bosutinib	Autophagy; p38 MAPK
Alisertib	Aurora Kinase; Autophagy
SB-590885	Autophagy; Ribosomal S6 Kinase (RSK)
Linrodostat	Apoptosis; ROCK
BAY 11-7085	Antibiotic; Autophagy; Bacterial
Lenvatinib	Apoptosis; Bcr-Abl; Src
GSK2837808A	Wee1
Telaglenastat	Apoptosis; Autophagy; Deubiquitinase; IKK
Tie2 kinase inhibitor 1	AMPK
Telatinib	Apoptosis; Autophagy; JAK; Mitophagy
Dinaciclib	Autophagy; Checkpoint Kinase (Chk)
CB30865	Apoptosis; MMP
Odanacatib	Apoptosis; Autophagy; mTOR
BAY 11-7082	Apoptosis; Autophagy; Topoisomerase
Tazemetostat	Histone Methyltransferase
Opaganib	SphK
Amuvatinib	Apoptosis; c-Kit; c-Met/HGFR; FLT3; PDGFR; RAD51; RET

Pelitinib	EGFR; Src
Gilteritinib	FLT3
IACS-6274	glutaminase
SN-38	TOP1
